# MXene (Ti_3_C_2_T_x_)/Cellulose Acetate Mixed-Matrix Membrane Enhances Fouling Resistance and Rejection in the Crossflow Filtration Process

**DOI:** 10.3390/membranes12040406

**Published:** 2022-04-06

**Authors:** Reem S. Azam, Dema A. Almasri, Radwan Alfahel, Alaa H. Hawari, Mohammad K. Hassan, Ahmed A. Elzatahry, Khaled A. Mahmoud

**Affiliations:** 1 Qatar Environment and Energy Research Institute (QEERI), Hamad Bin Khalifa University (HBKU), Qatar Foundation, Doha P.O. Box 34110, Qatar; ra1203108@student.qu.edu.qa (R.S.A.); delmasri@hbku.edu.qa (D.A.A.); 2 Department of Civil and Architectural Engineering, Qatar University, Doha P.O. Box 2713, Qatar; ra1404482@student.qu.edu.qa (R.A.); a.hawari@qu.edu.qa (A.H.H.); 3 Center for Advanced Materials, Qatar University, Doha P.O. Box 2713, Qatar; mohamed.hassan@qu.edu.qa; 4 Materials Science and Technology Program, College of Arts and Sciences, Qatar University, Doha P.O. Box 2713, Qatar; aelzatahry@qu.edu.qa

**Keywords:** membrane filtration, crossflow, MXene, antifouling

## Abstract

Obstacles in the membrane-based separation field are mainly related to membrane fouling. This study involved the synthesis and utilization of covalently crosslinked MXene/cellulose acetate mixed matrix membranes with MXene at different concentrations (CCAM-0% to CCAM-12%) for water purification applications. The membranes’ water flux, dye, and protein rejection performances were compared using dead-end (DE) and crossflow (CF) filtration. The fabricated membranes, especially CCAM-10%, exhibited high hydrophilicity, good surface roughness, significantly high water flux, high water uptake, and high porosity. A significantly higher flux was observed in CF filtration relative to DE filtration. Moreover, in CF filtration, the CCAM-10% membrane exhibited 96.60% and 99.49% rejection of methyl green (MG) and bovine serum albumin (BSA), respectively, while maintaining a flux recovery ratio of 67.30% and an irreversible fouling ratio at (*R_ir_*) of 32.70, indicating good antifouling performance. Hence, this study suggests that covalent modification of cellulose acetate membranes with MXene significantly improves the performance and fouling resistance of membranes for water filtration in CF mode relative to DE mode.

## 1. Introduction

Pressure-driven membrane filtration processes, such as microfiltration (MF), ultrafiltration (UF), nanofiltration (NF), and reverse osmosis (RO) are particularly promising solutions for clean water production and have attracted significant attention from researchers over the past few decades [1,2]. Many large-scale water treatment facilities prefer membrane technologies for water treatment due to their superior advantages of lower overall energy cost and ease of operation [3]. However, fouling is a chronic issue in membranes, which leads to the shortening of membrane lifespan and increases operational costs [1]. Recent work has concentrated on improving nanocomposite hydrophilic membranes for sustainable water treatment, targeting improvement in antifouling properties, and overcoming the trade-off between solution rejection and permeability [4].

In comparison to other hydrophilic polymers, cellulose acetate (CA) is mostly used to fabricate NF membranes [5,6,7] and is considered a promising substitute due to its outstanding film-forming capability, hydrophilic nature, biodegradability, superior toughness, tremendous biocompatibility, simple chemical modification, and fairly cheap price [8]. However, CA membranes are very prone to biological and organic fouling, which may result in the overall weakening of CA membranes’ separation performance [9]. This has inspired researchers to enhance the fouling resistance of CA membranes by the incorporation of additives, such as nanoparticles (NPs) [10] and 2D class materials, to attain enhanced separation performance [9,11]. 

Recently, polymer-based nanocomposite membranes have attracted significant awareness among other nanocomposites [12]. These are produced through the incorporation of NPs, nanofibers, or nanosheets/nanotubes into the membrane polymer matrix using different methods. However, nanoparticle incorporation into polymeric matrices results in their weak adhesion to the matrix as well as their aggregation [13]. For instance, Duval et al. [14], demonstrated poor adhesion between the polymer phase and the filler phase during the fabrication of zeolite-filled glassy polymer membranes. Furthermore, large NP contents weaken the membrane’s mechanical stability. The challenging and costly fabrication of nanoparticle-incorporated polymeric membranes can be avoided by using mixed-matrix membranes (MMMs). These can enable standard membranes to be used to address manufacturing process concerns [15]. The incorporation of 2D nanomaterials has enabled exceptional advancements in the preparation of MMMs due to their physicochemical properties that allow for ultrafast transport of water molecules, as well as their ability to provide surface functional groups that further enable membrane tunability [16]. 

Two dimensional (2D) metal carbides, MXenes, are derived from MAX phases, (M_n+1_AX_n_) where n: 1, 2 or 3 and M denote early transition metal elements, while A typically denotes non-metals from group IV-A, or III-A elements via etching whereby the A element-sheets are eliminated [17,18,19]. Ultimately, MXenes, or M_n+1_X_n_T_x_, are terminated by extremely hydrophilic and reactive T_x_ surfaces where T denotes O, OH, and/or F groups. Ti_3_C_2_T_x_ is the most investigated MXene in water treatment and environmental sanitizing applications, including for the elimination of ions, dyes, proteins, and heavy metals, among others [20]. MXenes have gained wide attention due to their distinctive properties, such as mechanical durability, good thermal and chemical stability, high surface area, favorable antifouling properties, high hydrophilicity (ready dissolution in water), metallic electronic conductivity, and film-making capability [21]. Our group has fabricated the first lamellar MXene membrane on PVDF using a vacuum-assisted filtration technique and used it for the rejection of multivalent cations from water [22]. Moreover, MXene (Ti_3_C_2_T_x_) flakes can be loaded on polymers to fabricate multifunctional films with appealing mechanical and electrochemical properties, outstanding conductivities, controlled thicknesses, and exceptional flexibility [23]. MXenes have fouling resistance ability and reveal high selectivity concerning different types of charged metal and dyes of different sizes and can resist cations with radiation radii greater than the interlayer spacing. 

Consequently, research has focused on the fabrication of mixed-matrix MXene composite membranes with various polymeric matrices (e.g., PES, PVA, PVDF) to overcome the constraints of neat MXenes, such as membrane swelling and loss of durability [24]. In our previous work, chemically crosslinked MXene-CA nanoporous mixed-matrix membranes were prepared via phase inversion followed by crosslinking with formaldehyde [18]. In the same work, our group studied the impact of chemical crosslinking and the loading of MXene on membrane efficiency in terms of permeation flux and rejection in comparison with non-crosslinked membranes The results confirmed that chemically crosslinked membranes with 10% MXene loading (CCAM-10%) exhibited a high pure water flux of ~256.9 LMH, 123.3% water uptake, and 69.7% porosity, together with more than 92.0% and 98.0% rejection of rhodamine B (RhB) and methyl green (MG), respectively [18]. However, these experiments were carried out in a stirred dead-end (DE) filtration setup. The disadvantage of DE filtration is usually the concentration polarization as well as fouling caused by the perpendicular flow of the feed fluid. 

Herein, chemically crosslinked MXene (Ti_3_C_2_T_x_)/CA mixed matrix filtration membranes were fabricated and their performance in flux, fouling resistance, as well as their separation efficiency, was tested in crossflow (CF) filtration mode to resemble the practical industrial setup. The effect of MXene loading in the CA matrix on the physicochemical properties, as well as the performance of the membrane in CF mode, is also presented. The turbulence produced across the surface of the membrane during CF filtration offers optimal permeation flux performance and extends filter functionality. Furthermore, since CF filtration is generally applied in membrane processes, information from CF filtration experiments offers flux and rejection performances that are appropriate to the industrial scale [25]. 

## 2. Materials and Methods

### 2.1. Materials

MAX (Ti_3_AlC_2_) was purchased from Y-Carbon, Ltd. Polyethylene glycol 400 (PEG-400), hydrochloric acid (HCl), acetone, acetic acid (≥34.5 wt.%), and sulfuric acid H_2_SO_4_ (98.8%), were purchased from Merck. CA (average Mn ~30,000), lithium fluoride (LiF) (99.0% F), formaldehyde (CH_2_O) ≥34.5 wt.%, bovine serum albumin (BSA), and MG (Methyl green) were purchased from Sigma-Aldrich. 

### 2.2. Fabrication of the MXene-CA Membrane 

Delaminated MXene (DL-MXene) sheets were prepared using the previously described procedure with some minor adjustments [18,26]. The MXene-CA nanoporous membranes with a mixing ratio of DL-MXene (0–12 wt.%) were prepared via the phase inversion method followed by formaldehyde (CH_2_O) crosslinking in 1:1 acetone/acetic acid solution following the procedure established by our group [18]. Afterwards, 0.5 g of PEG-400 was gently added, and the mixture was sonicated in a bath sonicator for 1 h to ensure a homogenous distribution of the MXene powder. To the homogenous mixture, 1.5 g of CA powder was added, and the mixture was stirred at 25 °C for 24 h. The solution was kept in a vacuum desiccator for 24 h to ensure no trapped bubbles were inside the solution. The viscous solution was poured onto a glass plate and, by using a casting knife film applicator (Elcometer 3580) and a Labcoat Master casting system (PHILOS, Gyeonggi-do, Korea), a thin film with a thickness of 280 μm was applied on the clean glass plate. The thin-film membranes were left to dry for 30 min at room temperature and then submerged in a coagulation bath consisting of cold DI water (~15 °C) for 2 h. Finally, the membranes were rinsed with DI water to remove any possible solvent residuals on the membrane surface. The prepared membranes were denoted as neat CA crosslinked membranes (CCAM) and MXene crosslinked CA membranes (CCAM-X%), whereby X% refers to the weight ratio of MXene to CA in the prepared membrane. A schematic representation of the phase inversion process for the fabrication of the crosslinked membranes is provided in Figure S1. 

### 2.3. Characterization

A powder X-ray diffractometer (XRD) was used to obtain the XRD data using a Bruker D8 Advance (Bruker AXS, Germany) with Cu/Ka radiation (λ = 1.5406 Å) at a current of 15.0 mA, a voltage of 40.0 kV, a scanning speed of 1°/min, and a step scan of 0.02°/step. Scanning electron microscopy (SEM) (FEI Quanta 650 FEG SEM) was used to study the morphology of the casted membranes and EDS mapping analysis of the prepared membranes was conducted at a voltage of 15 kV. A UV–vis spectrophotometer (Jasco V-670 absorption spectrophotometer at a scan speed of 100 nm${\;\min}^{-1}$) was used to measure the amount of methyl green (MG) and bovine serum albumin (BSA) in the feed and permeate solutions. Water contact angles were measured using a Rame-Hart contact angle goniometer equipped with a video camera and an image analysis system. The BET surface area and porosity were measured using an ASAP 2420 instrument with nitrogen adsorption and the measurements were calculated based on relative pressure. The surface topologies of the fabricated membrane were described with regards to surface roughness/morphology using an Asylum Research MFP-3D Origin+ atomic force microscope (AFM). The water uptake (WU) of the membranes was determined based on the dry weight of the membrane (under vacuum) and the weight of the membrane after being immersed for 24 h in DI water. 

### 2.4. Filtration Experiments

#### 2.4.1. Dead-End Filtration 

The separation performance of the fabricated membranes was first assessed using a dead-end (DE) filtration setup (Figure S2) (HP4750 stirred cell, Sterlitech, WA, USA) with an effective filtration area of 9.60 cm^2^. Unless stated otherwise, the filtration tests were conducted at a pressure of 1 bar and were stirred at 800 rpm and performed in duplicates. The weight of the permeate after 1 h of filtration time was measured and the water flux ($J_{w}$) of the fabricated membrane was calculated using Equation (1):

(1) $$\;\;\;\;J_{w}=V/AtP$$

where *V* denotes the quantity (volume/mass) of permeation (L), A indicates the effective surface area (m^2^), *P* is the applied pressure in bar, and *t* denotes the time in hours of the prepared membranes.

A feed solution stream comprising 100 mg L^−1^ MG, and 150 mg L^−1^ BSA was utilized to calculate the membrane rejection. 

The rejection (*R*) equation (Equation (2)) for solutes was calculated as follows:

(2) $$R\;\left(\%\right)=\frac{C_{f}-C_{p}}{C_{f}}\times 100$$

where $C_{f}$ and $C_{p}$ denote the concentration of the feed and permeate solutes in mg/L, respectively. The effective pore diameter (*a*) of the prepared hybrid NF membranes was measured via the Ferry equation (Equation (3)), as shown below [27], using DE filtration:

(3) R=100[1−(1−ra)2]2

R indicates the solute rejection (%) and *r* refers to the diameter of the solute. The MWCO of the fabricated membrane is the molar mass of the solutes which are 90% rejected by the membrane. Plotting the rejection (%) of the solutes against their corresponding molar mass in daltons allows the MWCO to be calculated [28].

#### 2.4.2. Crossflow Filtration

**Figure 1:**
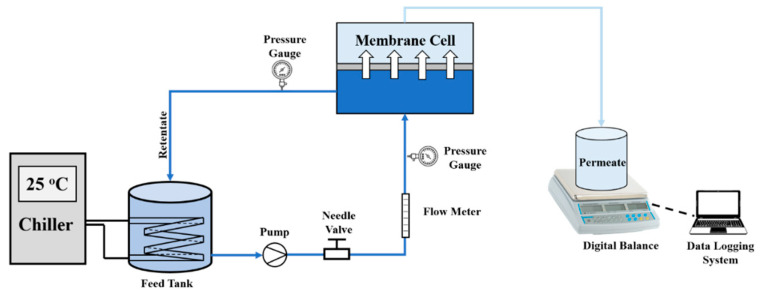
Crossflow filtration setup.

The separation performance of the prepared membranes was also evaluated using a crossflow filtration setup (Figure 1) which consisted of a chiller, a feed tank, a feed pump, a flow meter, a needle valve, a pressure gauge, a permeated collector, and a membrane module with an effective filtration area of about 42 cm^2^. The filtration tests were conducted at a pressure of 1 bar and the weight of the permeate was measured after 1 h of filtration time.

The rejection performance and pure water flux of the membranes were conducted and calculated following the procedure discussed in Section 2.4.1, using Equations (1) and (2).

### 2.5. Antifouling Evaluation

The membranes were tested with pure water at 1 bar for 30 min, then the flux was recorded as *J_W_*_0_ (${L\;m}^{-2}h^{-1})$. After that, the pure water was replaced with BSA solution consisting of 500 ppm and with pH 7 at 1 bar; the flux was recorded after 1 h (*J_p_* ($L{\;m}^{-2}h^{-1})$) and the rejection was analyzed and measured using UV. The membrane was washed with pure water for 30 min with no pressure and with the same CF velocity. Next, the membrane was operated at 1 bar with pure water for 30 min and the flux was recorded (*J_W_*_1_ (${L\;m}^{-2}h^{-1})$), indicating the end of one cycle. This was done to evaluate the antifouling property of CCAM-10%. Agglomeration of MXenes beyond 10 wt.% grafting was observed and found to negatively affect protein and dye rejection.

The flux recovery ratio (FRR) was characterized as follows (Equation (4)):

(4) $$\mathrm{FRR}=\left(\frac{JW1}{JW0}\right)\times 100\%$$

To accurately determine the fouling processes, resistance ratio measurements were applied to characterize the fouling resistance potential of the mixed matrix CCAM-10%. Resistance during the filtration process may indicate membrane fouling. The total fouling ratio was computed by applying the following equation (Equation (5)):

(5) $$R_{t}\left(\%\right)=\left(1-\frac{J_{p}}{J_{w0}}\right)\times 100\%$$

where $R_{t}$ denotes the total flux reduction, which is produced by the overall fouling and is known as the sum of the reversible fouling ratios; this describes the fouling resulting in concentration polarization. The irreversible fouling ($R_{ir}$) signifies fouling caused by BSA molecule adsorption onto the surface of the membrane; *R_r_* is the reversible fouling ratio. The below equations (Equations (6) and (7)) were used to calculate the reversible and irreversible fouling ratios, respectively [29].

(6) $$R_{r}\left(\%\right)=\left(\frac{J_{w1-}J_{p}}{J_{w0}}\right)\times 100\%$$

(7) $$R_{ir}\left(\%\right)=\left(1-\frac{J_{w1}}{J_{w0}}\right)\times 100\%=R_{t}-R_{r}$$

## 3. Results and Discussion

### 3.1. Surface Characterization of MXene/CA Membranes

**Figure 2:**
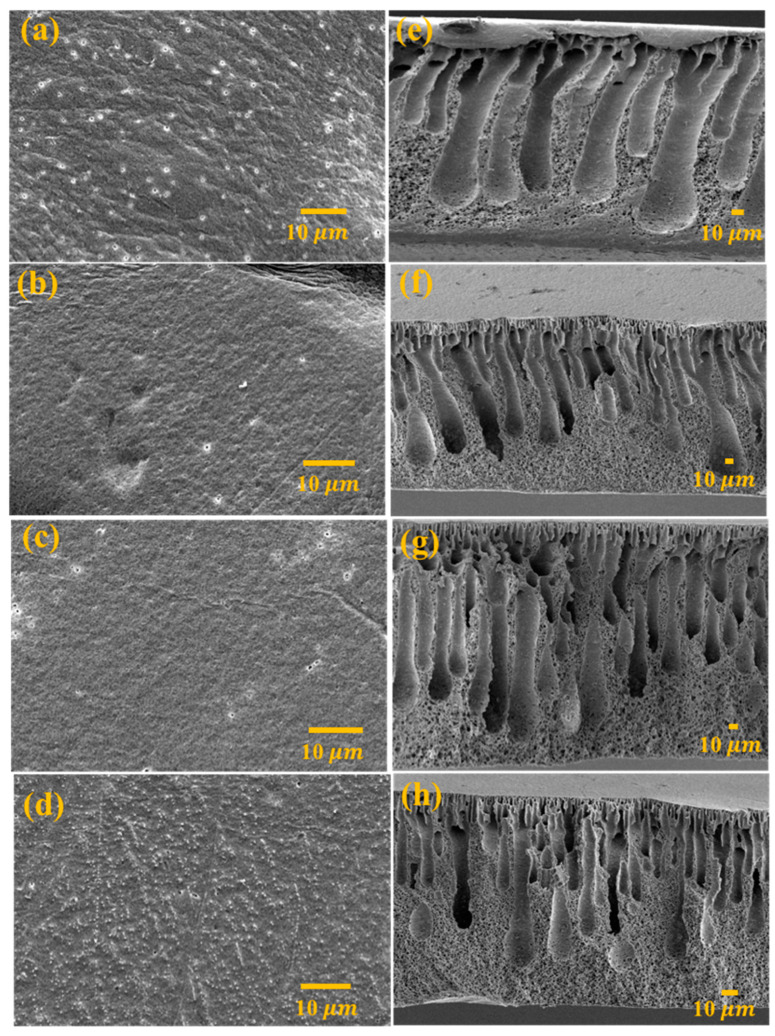
SEM pictures of the prepared membranes: (**a**) Surface CCAM-0% showing a dense structure; (**b**) surface CCAM-2%; (**c**) surface CCAM-8%; (**d**) surface CCAM-10% presenting a reduction in pore size after introducing MXene into the CA matrix; (**e**) cross-section of CCAM-0% displaying a dense structure; (**f**) cross-section of CCAM-2%; (**g**) cross-section of CCAM-8%; and (**h**) CCAM-10% cross-section showing a disordered channel arrangement.

SEM was used to study the surface morphology of the fabricated membranes, including the crosslinked CA membrane (CCAM-0%) and the crosslinked MXene/CA membranes (CCAM-X%), whereby X% denotes the wt.% of MXene in CA. The SEM images in Figure 2 depict the surface and cross-sectional areas of CCAM-0%, CCAM-2%, CCAM-8%, and CCAM-10% membranes. CCAM-0% revealed a dense structure with observed nanopores (Figure 2a,e). The surface SEM morphologies of CCAM (0–10%) indicated a homogeneous surface with no major surface defects (Figure 2a–d). The MXene (–OH and –O– surface-terminal groups) and CA (–OH group) contributed to forming a homogeneous structure that allowed for the uniform distribution of MXene in the CA polymeric matrix, leading to a smooth membrane surface morphology [18]. The homogenous dispersion and compatibility of the MXene 2D sheets in the CA polymer mixed matrix membrane were confirmed by the SEM (cross-section) images. All prepared membranes showed a comparable finger-like pore morphology due to the phase inversion method used for their fabrication [30,31]. Loading more MXenes into CCAM did not have a major effect on the finger-like pore configuration of CCAM-0% until CCAM-8% (Figure 2e–g). However, the surface and cross-sectional morphology of CCAM-10% showed a denser structure with more disordered channel arrangements, most likely due to the condensation of the MXene sheets in the channels and chemical cross-linking among MXene and CA [18,27]. 

EDS mapping (cross-section) confirmed the uniform dispersion of MXene into the CA membrane which was indicated by the distribution of titanium in the matrix (Figure S3a–c). The good dispersion of the MXene content into the composite CA membrane maintained its hydrophilicity, thereby generating a superior permeate flux [24]. Introducing MXene into CCAM was anticipated to improve membrane hydrophilicity and enhance membrane fouling resistance. 

**Figure 3:**
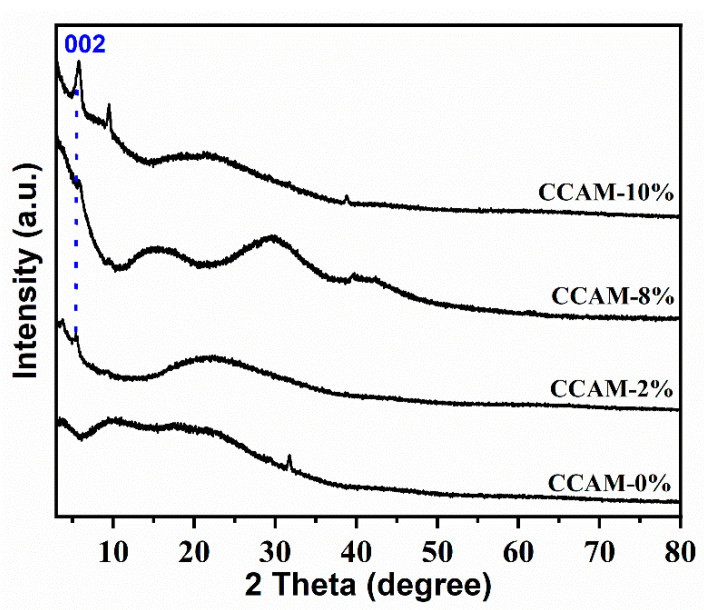
XRD of crosslinked cellulose acetate MXene membranes.

The CCAM-X% membranes were studied using XRD to determine the effect of the distribution of the 2D MXene sheets into the CCAM polymer matrix (Figure 3). CCAM-0% was characterized by (002) diffraction peaks at 2θ=~23.00° and 30.00°[18,32]. The (002) diffraction peak spotted at 2θ=~6.900° can be attributed to DL-MXene. The MXenes showed good interaction with CA due to the observed shift of the (002) MXene diffraction peak by 2° to a smaller angle after MXene loading [33]. This showed that membrane crystallinity was influenced by the amorphous nature of CA which caused this shift. Similarly, the (002) diffraction peak shift in the direction of smaller angles after introducing MXene content into the CA matrix was due to a greater dispersion of the MXene sheets along with the CA matrices. The formation of a Ti_3_C_2_T_x_ (MXene)/CA hybrid membrane was confirmed by the intercalation of the CA chains with the MXene sheets [23]. 

### 3.2. Hydrophilicity, Surface Area, and Morphology of CCAM and CCAM-X%

**Figure 4:**
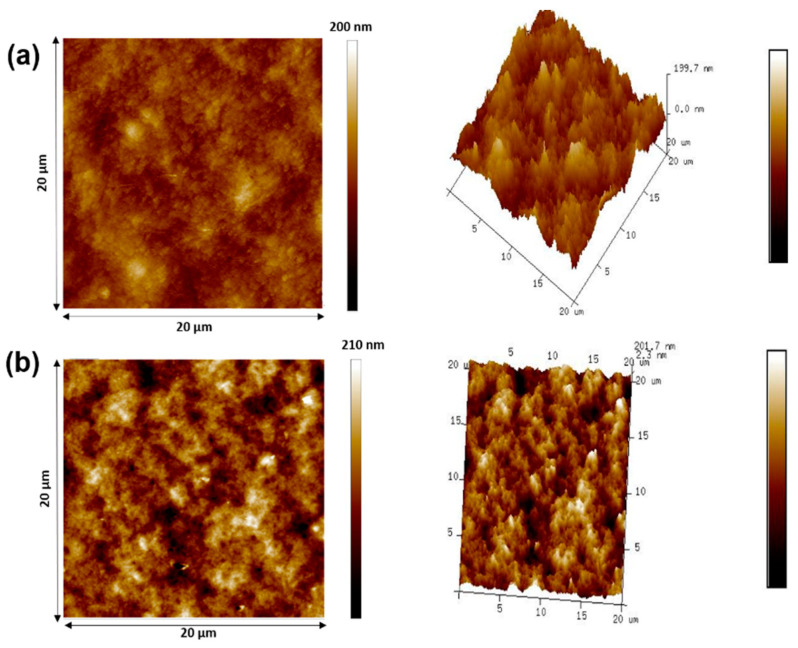
Atomic force microscopy (AFM) 2D and 3D images for (**a**) CCAM-0% and (**b**) CCAM-10%.

Atomic force microscopy (AFM) was utilized to inspect the surface roughness of the crosslinked mixed matrix composite membranes. In the AFM images (Figure 4), the lightest areas indicate the maximum point of the membrane surface, while the dark areas represent the lower topography or valleys of the membrane pores [34]. The membrane surface roughness changed due to the loading of MXene. While the surface roughness of CCAM-0% (pristine membrane) was virtually free of any significant roughness features [35], there were rough areas distributed uniformly across the surface of CCAM-10%, indicating the good compatibility of MXene with the CA mixed matrix [36]. 

The most common surface roughness parameters obtained from an AFM analysis are average roughness (Ra) and root square roughness (Rq) [37]. The surface roughness parameters of CCAM-0% and CCAM-10% are tabulated in Table 1. As shown in Figure 4 and Table 1, CCAM-10% (Ra = 47.40 and Rq = 60.20 (nm)) had a rougher surface than CCAM-0% (Ra = 22.50 and Rq = 28.40 (nm)) due to the hydrophilic nature of MXene, which may have induced a faster solvent and non-solvent exchange throughout the phase inversion process [38]. This indicated the excellent adherence of MXene 2D sheets to the membrane surface. The water contact angle decreased from 71.30° for CCAM-0% to 48.60° for CCAM-10% after the introduction of MXene into the CA membrane matrix, which indicated the good hydrophilic characteristic of MXene [18]. The membrane contact angles decreased as the membrane surface roughness increased (Figure S4). Increasing the surface roughness of a hydrophilic surface is known to enhance the wettability of the surface, hence, to decrease the surface’s contact angle [39].

Table 2 summarizes the BET specific surface area, average pore diameter, and pore volume values for CCAM-0% and CCAM-10% measured by nitrogen adsorption. The specific surface area of CCAM-0% was 44.27 m^2^/g and an almost three-fold increase in surface area was observed for CCAM-10% (124.3 m^2^/g). This significant increase indicated that the incorporation of MXene into the CA polymer matrix played an important role in improving the membrane surface area, thus, providing more membrane adsorption sites. A comparable increasing profile was observed for the membrane pore volumes. However, a dramatic decrease from 12.83 nm for CCAM-0% to 1.91 nm for CCAM-10% was observed in the membrane pore size while the porosity of the membranes was shown to increase by a ratio of 1.24. This trend was expected as MXene is a porous material, which enhances the porosity of the membrane. Furthermore, the formation of ionic clusters within the CCAM, due to strong hydrogen bond development, as well as the presence of void volumes, was directly proportional to the water uptake and porosity of the membrane [18].

The water uptake and porosity of CCAM-10% was found to be 125.3% and 72.3%, respectively, in our previous work [18]. Using (Equation (3)), the CCAM-10% effective mean pore diameter was computed and found to be ~1.730 nm. The MWCO of the CCAM-10% membrane, determined by the molar mass of the solutes that represented 90% of the membrane rejection, was calculated to be 435 daltons at a rejection of 90%. Hence, CCAM-10% was chosen as the optimal membrane composition, as CCAM-12% exhibited a lower rejection performance attributed to MXene agglomeration which will be discussed in a later section.

### 3.3. Membrane Separation Performance

Based on the above performance evaluation, CCAM-10% showed the best structural and surface morphology properties and was selected as the optimal membrane composition for subsequent analysis. To optimize the membrane performance, it is essential to examine the impact of the process parameters, such as the operating pressure, on the rejection. 

Laboratory-scale DE has been recognized as a facile experimental process and usually provides a good indication of membrane performance with lower energy consumption compared to other filtration techniques [40]. However, in most practical applications, the buildup of rejected species is very harsh, meaning DE filtration becomes unfeasible and a CF operation must be employed. In CF mode, the feed flow is tangential, which assists in removing the accumulated rejected particles or molecules at the surface of the membrane. This reduces the formation of cake layers and, thus, maintains a good permeation flux. The stirring action in the dead-end cell utilized here was intended to mimic the crossflow action by providing fluid movement tangential to the membrane surface. However, as the fluid volume in the dead-end cell decreased, this became less applicable with an increased solute concentration close to the membrane surface than experienced in CF. Furthermore, CF offers the benefit of improved membrane lifetime by reducing irreversible fouling [41]. In this work, first the stirred DE filtration process was used to evaluate the membrane water flux and rejection performance. Subsequently, the CF filtration mode was adopted to test the membrane separation and antifouling performance in terms of improved fouling resistance that can withstand severe environmental conditions. 

#### 3.3.1. Effect of Operating Pressure on Membrane Rejection

**Figure 5:**
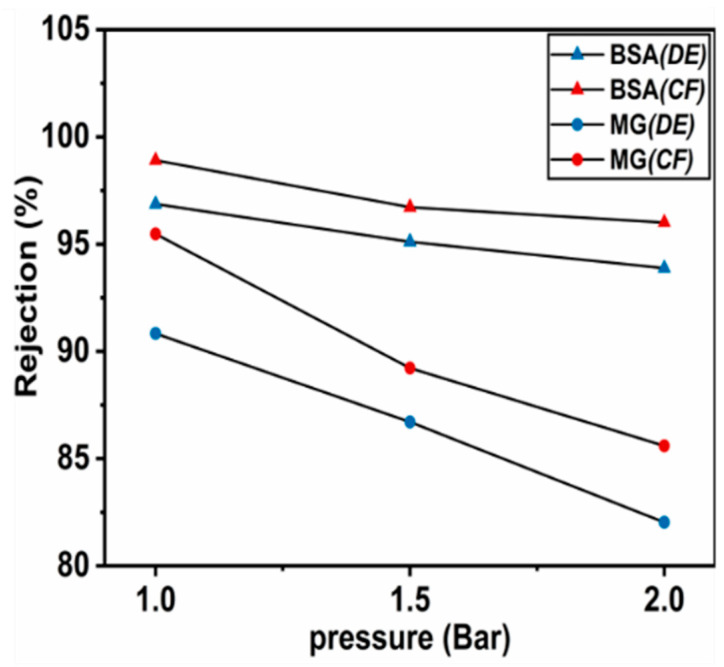
Effect of the operating pressure (1–2 bar) on the rejection of MG (100 ppm) and BSA (150 ppm) using DE (blue symbol) and CF (red symbol) filtration (CCAM-10%) for 1 h.

The impact of the operating pressure (1–2 bar) on the rejection of CCAM-10% using DE and CF filtration was tested. As shown in Figure 5, the rejection of both MG and BSA by CCAM-10% in CF filtration mode outperformed that of the DE filtration mode. In both filtration modes, the rejection of MG and BSA decreased with increasing operating pressure and BSA rejection was higher than that of MG. CCAM-10% showed the highest rejection of MG and BSA (96.60% and 99.49%, respectively) at 1 bar in CF filtration mode, which decreased to 86.72% and 96.59% for MG and BSA, respectively, when tested at 2 bar. In DE filtration mode, when the pressure was increased from 1 bar to 2 bar, the rejection of MG and BSA decreased from 92.13% to 83.32% and 97.97% to 94.98%, respectively. The lower rejection efficiency observed in DE filtration was attributed to the higher force applied on MG and BSA in the direction perpendicular to the membrane surface/pores, which could not be fully mitigated by the shearing action of the stirring, as was the case in CF filtration, which increased movement of the solute tangential to the membrane surface. At higher pressure, less MG and BSA was rejected in both filtration modes. The higher pressure increased the shear force in the direction of permeate flow, forcing more compounds to lose their hydration shells and pass through the membrane pores [42]. Due to the high rejection efficiencies of both MG and BSA obtained at 1 bar, this operating pressure was used for the remaining experiments. 

#### 3.3.2. Effect of MXene Loading on Membrane Permeation and Rejection

**Figure 6:**
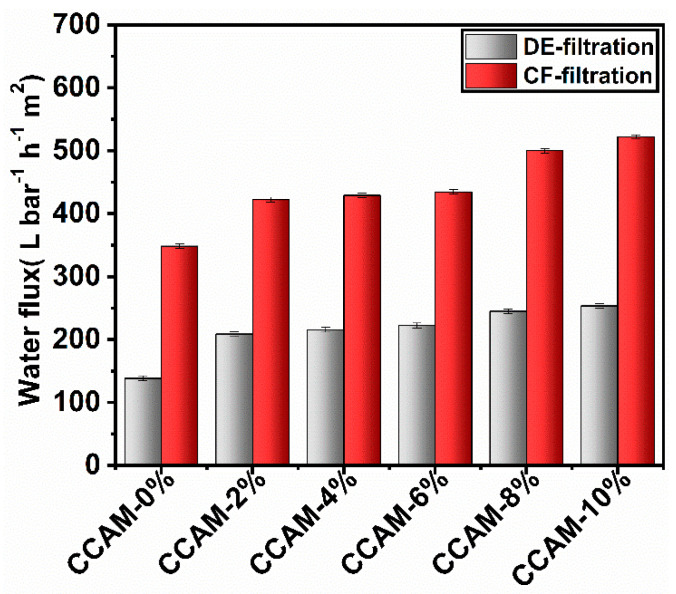
Water flux of CCAMs with various MXene contents (0–10%) for 1 h and 1 bar using DE and CF filtration.

The impact of MXene loading on the permeation flux using membranes in both CF and DE filtration modes were measured and plotted, as shown in Figure 6. Out of all the membranes (CCAM (0–10%)) tested using CF filtration, the highest water flux was observed for CCAM-10% (522.3 LMH). This was attributed to the hydrophilic nature of MXene and its consequential development of extra nanopores in the membrane [18]. Moreover, membranes with higher roughness offer a more open area for membrane transport; thus, the permeation flux was enhanced with the increased surface roughness obtained by the addition of MXene [43]. Higher surface roughness was expected to increase the specific surface area by providing a greater contact area for the foulants and improving the hydrophilicity of the membrane, thus increasing the feed water flux, as demonstrated in previous studies using dead-end filtration [44,45] and crossflow filtration [46]. 

As expected, the water flux resulting from CF filtration was higher than that resulting from DE filtration for all the prepared membranes. This was attributed to concentration polarization occurring on the surface of the membrane due to contaminant accumulation, hence, slowing down the membrane flux [42,47]. The water flux changes according to the membrane hydrophilicity, pore size, and density, whereas the effluent rejection is controlled by the pore size and surface charge of the membrane. This was clear from the results of the water flux in both the DE and CF modes (Figure 6). In the DE mode, the flux of CCAM-0% (138.4 LMH) was enhanced to 208.8 LMH and, in the CF mode, the flux of CCAM-0% (348.5 LMH) increased to 422.3 LMH after introducing a mere 2 wt.% of MXene (CCAM-2%). This provided further evidence that MXene increased the membrane hydrophilicity which was confirmed with the contact angle results (Figure S4). 

The rejection performance of CCAM-0%, CCAM-8%, and CCAM-10% using CF filtration was compared with MG (100.0 mg L^−1^) and BSA (150.0 mg L^−1^) solutes and tested at 1 h and 1 bar (Table 3). The performance of these membranes in CF filtration mode showed an increasing trend in rejection with increasing MXene content which could be attributed to the CH_2_O cross-linking as well as the creation of denser layers. The rejection using CF filtration was also higher than that of DE filtration. CCAM-10% was found to be the optimum membrane for the rejection of MG and BSA as studies with CCAM-12% in DE filtration mode showed lower dye and protein rejection (84.31% and 76.50% for MG and BSA rejection, respectively) which could be due to the agglomeration of MXenes beyond 10 wt.% grafting. As reported in our recent work [18], the rejection behavior of the fabricated membranes is a physical filtration phenomenon. 

The performance of the CCAM-10% membrane was compared, in terms of dye/protein rejection and pure water flux, to other membranes reported in the literature (Table 4). The overall performance of CCAM-10% exceeded or was similar to that for other reported membranes. 

### 3.4. Membrane Antifouling Performance

In this study, the CF filtration process was applied to analyze the antifouling effectiveness of the membranes. A typical protein (BSA) was selected to assess the antifouling ability of the CCAM-X% membranes. The best parameters for estimating the membrane antifouling properties are the flux recovery ratio (FRR), total fouling resistance ratio (*R_t_*), reversible fouling resistance ratio (*R_r_*), and the irreversible fouling resistance ratio (*R_ir_*). 

**Figure 7:**
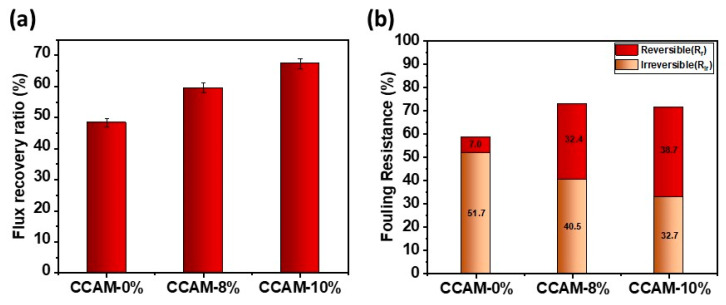
(**a**) Water recovery ratio of CCAM-0%, CCAM-8%, and CCAM-10% after fouling using 500 ppm BSA protein solution using CF filtration; (**b**) fouling resistance ratios of CCAM-0%, CCAM-8%, and CCAM-10% using CF filtration.

The FRR% of the prepared CCAM-0%, CCAM-8%, and CCAM-10% membranes is presented in Figure 7a. A higher percentage of FRR signifies better antifouling property of the membrane. Increasing the MXene loading improved the FRR%, indicating better fouling resistance performance. The fouling resistance performance of the membrane was most likely affected by the membrane hydrophilicity, and the increasing trend in FRR% matched the membranes’ contact angle values with increasing MXene content (Figure S4). The presence of the hydrophilic MXene 2D sheets induced the formation of a hydration layer on the surface of the membrane, which enabled stearic and energetic hindrance against organic foulant adsorption to the surface of the membrane [61,62]. Based on these results, it can be inferred that the incorporation of MXene nanosheets into the CCAM provided effective antifouling properties. 

Other important parameters related to membrane fouling, *R_r_* and *R_ir_*, were calculated and are presented in Figure 7b. The reversible fouling ratio played a major role in the overall fouling of CCAM-10% (38.70%); therefore, foulants trapped on the surface and in the membrane pores could be easily removed by washing with DI water. The irreversible ratio was observed to decrease from 51.7% (CCAM-0%) to 40.50% (CCAM-8%), and finally to 32.70% (CCAM-10%). These results confirmed that CCAM-10% demonstrated significant fouling resistance in comparison to CCAM-0% and CCAM-8%. This enhancement could be ascribed to the more hydrophilic nature and negative surface charge of CCAM-10%, which played a fundamental role in resisting the foulants, preventing them from being continuously adsorbed to the membrane surface [63]. 

The improved membrane surface properties, including hydrophilicity and charge, and the subsequent reduction in membrane fouling in CCAM-10%, can be ascribed solely to the successful incorporation of 10 wt.% MXene into the CCAM [61,62]. The reported enhanced separation performance of the CCAM-10% membrane was mainly attributed to the significant physicochemical properties of the incorporated MXene. The hydrophilic MXene nanosheets acted as porous nanofillers that facilitated the passage of water molecules through the membrane, thereby, enhancing the pure water flux. The contaminant rejection is controlled by the pore size and surface charge of the membrane and, as demonstrated here, as well as in our previous study [26], the chemical crosslinking effect and addition of MXenes in CA, decreased the membrane pore size and increased the porosity. This characteristic enabled the dye/protein to be rejected via pore size exclusion. 

## 4. Conclusions

This study demonstrated the ability of MXene/CA composite membranes, particularly CCAM-10%, to exhibit very high permeation flux and excellent anti-fouling resistance properties in CF filtration mode relative to DF filtration. CCAM-10% was demonstrated to be the most hydrophilic and shown to cause the least membrane fouling. In contrast to the DE filtration configuration, the CF tangential flow filtration process prevented cake accumulation on the membrane surface. This was demonstrated by the performance of CCAM-10% in CF filtration mode which exhibited a high permeation flux of 522.3 LMH, a more than 43% increase relative to DE filtration. CF filtration resulted in higher rejection of MG (96.60%) and BSA (99.49%) when compared to DE filtration (92.13%, MG; 83.32%, BSA) for CCAM-10%. Furthermore, when tested in CF filtration mode, CCAM-10% exhibited high FRR% and *R_r_* values of 67.3% and 38.7%, respectively, and a low *R_ir_* value of 32.7%. The CCAM-10% membrane could be used in various water treatment applications, including CF filtration. This is based, in part, on the fouling resistance, excellent physicochemical properties, and separation performance of the MXene @ CA composite membrane, which provides a basis for efficient and highly profitable UF/NF membranes based on MXene CCAMs. 

## Figures and Tables

**Table 1:** Surface roughness parameters of CCAM-0% and CCAM-10%.

Membrane	CCAM-0%	CCAM-10%
Average roughness (Ra (nm))	22.50	47.40
Root square roughness (Rq (nm))	28.40	60.20

**Table 2:** BET surface area, mean pore diameter, and pore volume of CCAM-0%, and CCAM-10%.

Membrane	Specific Surface Area (m^2^/g)	Mean Pore Diameter (nm)	$\mathrm{Adsorption}\;\mathrm{Micro}-\mathrm{Porous}\;\mathrm{Volume}\;(cm^{3}/g)$
CCAM-0%	44.27	12.83	0.284
CCAM-10%	124.3	1.910	0.781

**Table 3:** A comparison between rejection of MG and BSA by CCAM-0%, CCAM-8%, and CCAM-10% using DE filtration and CF filtration for 1 h and 1 bar.

Membrane	Rejected Dye/Protein	Rejection (%), Dead-End	Rejection (%), Crossflow
CCAM-0%	MG	28.91	49.70
	BSA	73.00	82.75
CCAM-8%	MG	79.90	80.33
	BSA	90.80	97.23
CCAM-10%	MG	92.13	96.60
	BSA	97.97	99.51

**Table 4:** Performance comparison amongst recently reported literature on CA, MMM, MWCNT and GO incorporated membranes.

Membrane	Dye/Protein	Water Flux (L m^−2^h^−1^bar^−1^)	Rejection (%)	Reference
M-PES/ZIF-67	BSA	~56.00	98.00	[48]
MOFs UiO-66 NH_2_-PES-MMM	BSA	300.0	95.00	[49]
PET-PEG3	BSA	~12.00	94.00	[50]
PVDF/PFSA	BSA	~461.0	88.00	[51]
TFMGs/W-PSF_10_	BSA	~322.0	99.90	[52]
CA/E-WS_2_(1 wt.%)	BSA	~107.0	~97.00	[53]
PES-CA	BSA	~63.00	~ 85.00	[54]
PES-CA-Ag_2_O		~93.00	~89.00	
ZrO_2_/BCM	BSA	~322.0	~91.00	[55]
18 wt.% PVC Hollow fiber	MG	~32.00	75.20	[56]
CA/TiSiO_4_ (20 wt.%)	BSA	134.0	98.80	[57]
PSF/MXene	BSA	306.0	98.00	[34]
PSF/SiO_2_GO	BSA	376.0	98.00	[58]
PVDF/GO	BSA	243.0	~77.00	[59]
ZCA	BSA	~137.0	~98.00	[60]
MXene (Ti_3_C_2_T_x_)	MG	118.0	94.00	[26]
	BSA		100.0	
21% Ag at MXene	MG	420.0	92.00	
	BSA		100.0	
CCAM-10%	MG	522.3	96.60	This Work
	BSA		99.50	

## Supplementary Materials

The following supporting information can be downloaded at: https://www.mdpi.com/article/10.3390/membranes12040406/s1, Figure S1: Schematic representation of the phase inversion of MXene in cellulose acetate membrane; Figure S2: Dead-end filtration process setup; Figure S3: EDS cross-section mapping analysis images for (a) CCAM-0%, (b) CCAM-2%, (c) CCAM-8%, and (d) CCAM-10%; Figure S4: Water contact angle of cross-linked CCAM-X% with MXene contents (X) from 0% to 10%.
