# Supplementary material for: MXene (Ti3C2Tx)/Cellulose Acetate Mixed-Matrix Membrane Enhances Fouling Resistance and Rejection in the Crossflow Filtration Process
Source: Membranes (Basel). 2022 Apr 6;12(4):406. doi: 10.3390/membranes12040406 (PMC9027356; doi:10.3390/membranes12040406)
Supplement: Supplementary file 1 [file membranes-12-00406-s001.zip › membranes-1632238-supplementary.pdf]

membranes

MDPI

Supporting Information

MXene ($Ti_3C_2T_x$)/ cellulose acetate mixed-matrix membrane enhances the fouling resistance and rejection in the crossflow filtration process

Reem S. Azam [a], Dema A. Almasri,[a] Radwan Alfahel[b], Alaa H. Hawari[b,], Mohammad K. Hassan[c], Ahmed A. Elzatahry[d] and Khaled A. Mahmoud [a,c,*].

[a] Qatar Environment and Energy Research Institute (QEERI), Hamad Bin Khalifa University (HBKU), Qatar Foundation, P.O. Box 34110, Doha
[b] Department of Civil and Architectural Engineering, Qatar University, P.O. Box 2713, Doha, Qatar.
[c] Center for Advanced Materials, Qatar University P.O. Box 2713, Doha, Qatar.
[d] Materials Science and Technology Program, College of Arts and Sciences, Qatar University, Doha, 2713, Qatar

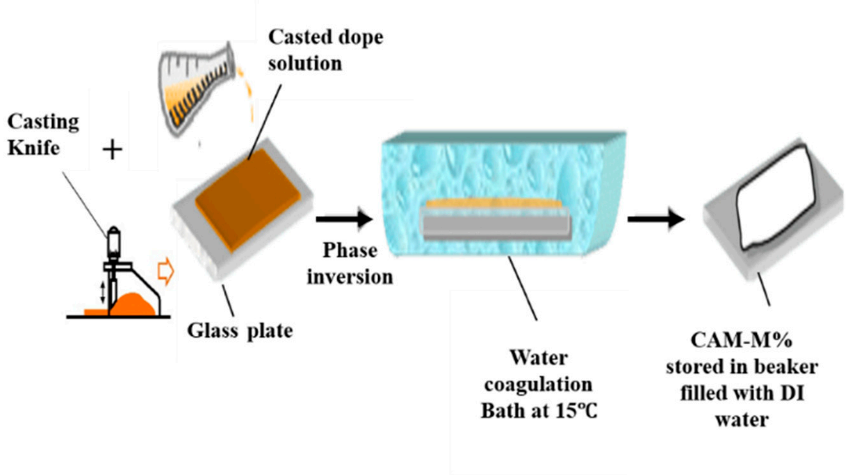

Figure S1. Schematic representation of the phase inversion of MXene in cellulose acetate membrane.

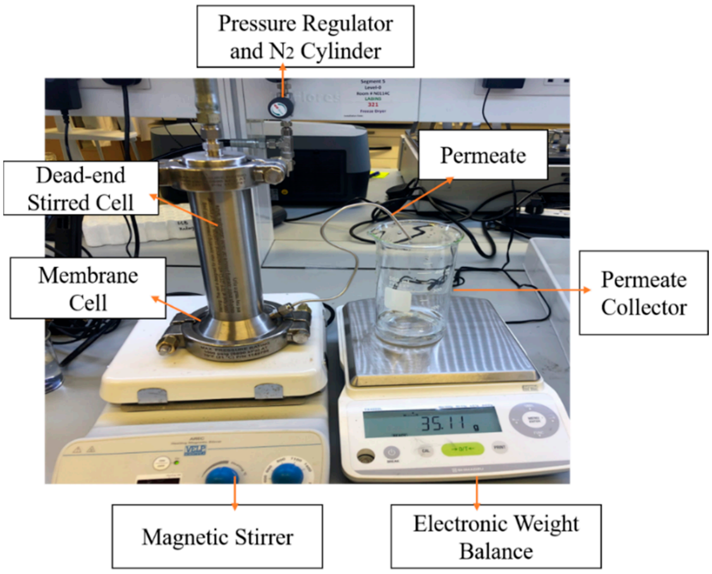

Figure S2. Dead-end filtration process setup.

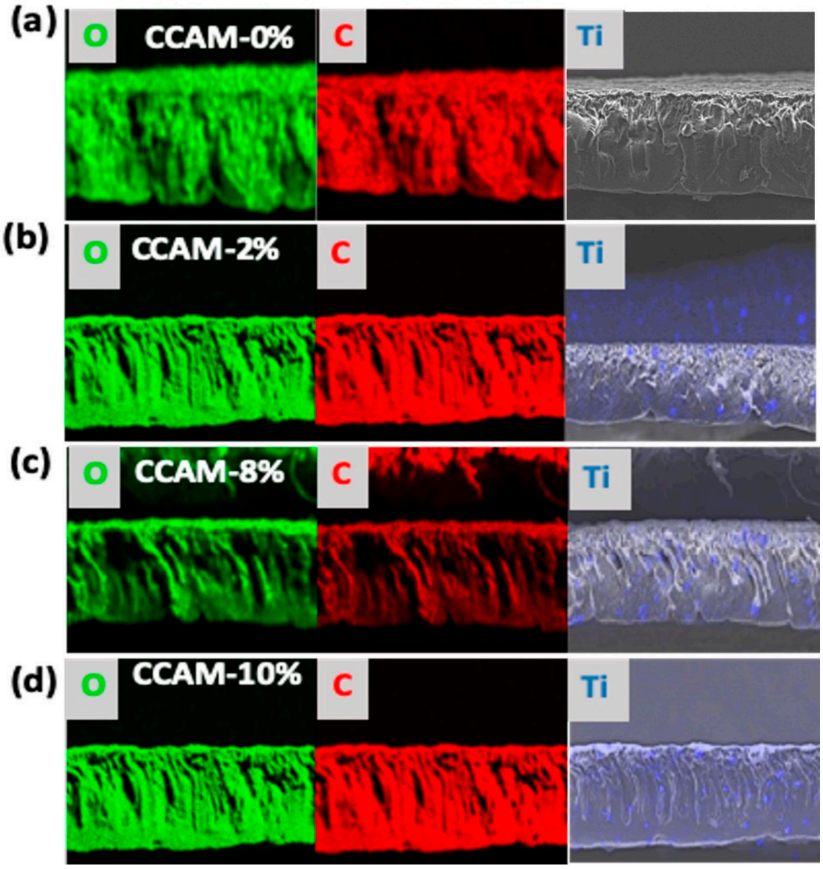

Figure S3. EDS cross-section mapping analysis images for (a) CCAM-0%, (b) CCAM-2%, (c) CCAM-8%, and (d) CCAM-10%.

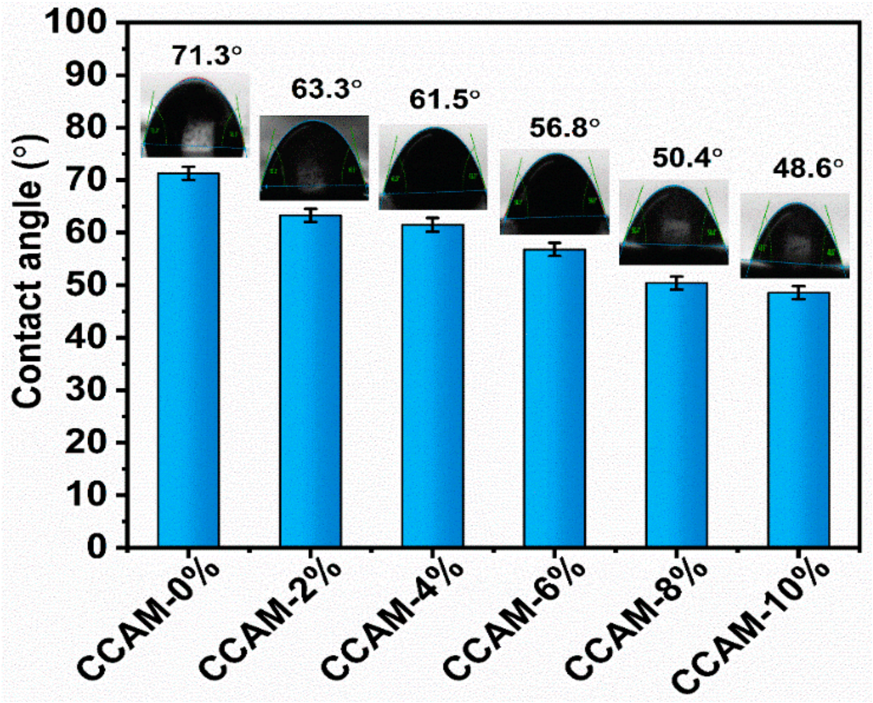

Figure S4. Water contact angle of cross-linked CCAM-X% with MXene contents (X) from 0% to 10%.
